# ExpressHeart: Web Portal to Visualize Transcriptome Profiles of Non-Cardiomyocyte Cells

**DOI:** 10.3390/ijms22168943

**Published:** 2021-08-19

**Authors:** Gang Li, Changfei Luan, Yanhan Dong, Yifang Xie, Scott C. Zentz, Rob Zelt, Jeff Roach, Jiandong Liu, Li Qian, Yun Li, Yuchen Yang

**Affiliations:** 1 Department of Statistics and Operations Research, University of North Carolina, Chapel Hill, NC 27599, USA; franklee@live.unc.edu; 2 Department of Biostatistics, University of North Carolina, Chapel Hill, NC 27599, USA; luanchangfei6@gmail.com (C.L.); zentz@email.unc.edu (S.C.Z.); 3 Department of Pathology and Laboratory Medicine, University of North Carolina, Chapel Hill, NC 27599, USA; yanhande@email.unc.edu (Y.D.); yfxie@email.unc.edu (Y.X.); jiandong_liu@med.unc.edu (J.L.); li_qian@med.unc.edu (L.Q.); 4 McAllister Heart Institute, University of North Carolina, Chapel Hill, NC 27599, USA; 5 Research Computing, University of North Carolina, Chapel Hill, NC 27599, USA; robz@email.unc.edu (R.Z.); jeff_roach@unc.edu (J.R.); 6 Department of Genetics, University of North Carolina, Chapel Hill, NC 27599, USA; 7 Department of Computer Science, University of North Carolina, Chapel Hill, NC 27599, USA

**Keywords:** non-cardiomyocytes, single-cell RNA-sequencing, expressheart, R Shiny, visualization, differentially expressed genes, cross-species comparison

## Abstract

Unveiling the molecular features in the heart is essential for the study of heart diseases. Non-cardiomyocytes (nonCMs) play critical roles in providing structural and mechanical support to the working myocardium. There is an increasing amount of single-cell RNA-sequencing (scRNA-seq) data characterizing the transcriptomic profiles of nonCM cells. However, no tool allows researchers to easily access the information. Thus, in this study, we develop an open-access web portal, ExpressHeart, to visualize scRNA-seq data of nonCMs from five laboratories encompassing three species. ExpressHeart enables comprehensive visualization of major cell types and subtypes in each study; visualizes gene expression in each cell type/subtype in various ways; and facilitates identifying cell-type-specific and species-specific marker genes. ExpressHeart also provides an interface to directly combine information across datasets, for example, generating lists of high confidence DEGs by taking the intersection across different datasets. Moreover, ExpressHeart performs comparisons across datasets. We show that some homolog genes (e.g., *Mmp14* in mice and *mmp14b* in zebrafish) are expressed in different cell types between mice and zebrafish, suggesting different functions across species. We expect ExpressHeart to serve as a valuable portal for investigators, shedding light on the roles of genes on heart development in nonCM cells.

## 1. Introduction

Heart disease is a major cause of morbidity and mortality, leading to more than 8.8 million deaths in 2019 (World Health Organization, 2019). A comprehensive interrogation and visualization of the cell composition in the heart and their corresponding functions will provide important clues to the development of therapeutic strategies for heart diseases. Existing research has focused primarily on cardiomyocytes (CMs), to understand the underlying mechanisms of the cardiac response to heart failure [1,2,3,4,5]. However, the heart, as a heterogeneous organ, contains several other important cell types besides CMs, including fibroblasts, endothelial cells, and a wide variety of immune cells. Serving more than bystanders of cardiac function, these non-cardiomyocytes (nonCMs) have been found to play critical roles in providing structural, mechanical, and electrophysiological support to the working myocardium [6,7]. However, our knowledge of the molecular features of nonCMs remains limited.

Single-cell RNA sequencing (scRNA-seq) has emerged as a powerful tool to dissect transcriptional profiles of complex tissues at single-cell resolution. Compared to traditional bulk RNA sequencing technologies, scRNA-seq identifies and characterizes sub-cell types/intermediate cellular states, even for rare cell types/cellular states with a limited number of cells. In recent years, increasingly, more studies have employed scRNA-seq or single-nucleus RNA-seq (snRNA-seq) to investigate the transcriptional features of the nonCM populations, broadening our knowledge on the molecular functions of the nonCM cells [8,9,10,11,12,13,14,15,16,17,18,19,20]. For example, Skelly et al. employing scRNA-seq, comprehensively characterized gene expression profiles of nonCMs in mouse hearts at a single-cell level, which provides a comprehensive view of the diversity and specific molecular features, as well as intercellular communication, for different nonCM cell types [9]. For another example, Wang et al. characterized the transcriptional landscape of three fibroblast subtypes in different functional states from adult murine hearts [18]. These three subtypes of fibroblast cells participate in cellular response, cytoskeleton organization, and immune response, respectively. McLellan et al. found that fibroblasts with *Clip* expressed, which they referred to as Fibroblast-*Cilp*, emerge as the most abundant fibroblast sub-population in response to pathological remodeling of the heart [14]. Using snRNA-seq data, Nicin et al. showed age-dependent transcriptional dynamics in fibroblasts in pediatric patients with dilated cardiomyopathy [19]. These findings not only shed light on molecular signatures and cellular functions of nonCMs, but also provide valuable resources for future studies. However, although most published scRNA-seq data of nonCMs are publicly available, there is no existing platform allowing researchers to easily visualize or analyze these cardiac-related datasets for nonCM cells. Moreover, the published scRNA-seq data encompass different species (humans, mice, and zebrafish) and under different conditions (e.g., healthy heart or heart having experienced a myocardial infarction (MI)). Thus, integrating these datasets will allow joint analysis and visualization. For instance, across-dataset comparisons enabled by a centralized platform will provide an improved understanding of conservation across species and differentiation in molecular signatures underlying a certain biological process, which may shed light on the therapeutic transformation. However, such a platform is still missing in the field.

Here, we present an open-access web service, ExpressHeart (http://shiny.bios.unc.edu/expressheart/), for visualizing or analyzing scRNA-seq datasets of nonCMs of humans, mice, and zebrafish derived from five studies (detailed in Results Section 2.1). We expect ExpressHeart to serve as a valuable portal for investigators studying nonCMs, shedding light on the roles of genes on heart development in various nonCM cells.

## 2. Results

### 2.1. Overview of ExpressHeart

**Figure 1:**
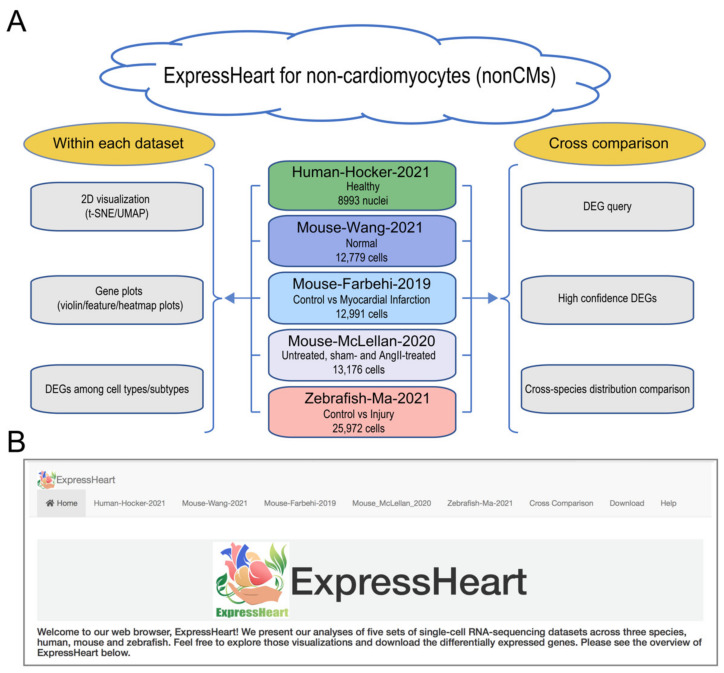
Overview of ExpressHeart. (**A**) Framework and (**B**) interface of ExpressHeart. ExpressHeart contains five scRNA-seq or snRNA-seq datasets (middle of panel A). These datasets are named by the last name of the first author, species, and the year of the corresponding publication or data release. The conditions and numbers of cells/nuclei are also presented in each dataset. On the left of panel A, we show three types of visualization and/or analysis within each dataset, including 2D visualization, gene plots via violin/feature/heatmap, and DEGs among cell types/subtypes. On the right of panel A, we show three types of visualization and/or analyses across different datasets. We provide a module (“Cross-comparison”) for DEG query across all datasets, where we report high confidence DEGs across multiple datasets within the same species for major cell types and compare homolog gene distribution across different species. Panel B shows the interface of the web portal. Each dataset has its own analysis and visualization module, using the same names specified in panel A. “Cross-comparison” module corresponds to three sub-modules showed in the right of panel A.

The goal of our web portal ExpressHeart is to provide researchers a user-friendly platform to visualize and analyze cellular and molecular features of cardiac nonCMs using publicly available datasets (Figure 1A). ExpressHeart currently incorporates five scRNA-seq datasets from three species, namely, Human-Hocker-2021 (healthy) [17], Mouse-Wang-2021 (wildtype) [18], Mouse-Farhebi-2019 (control (mice undergoing surgical incision without ligation of the left anterior descending coronary artery) vs. MI) [11], Mouse-McLellan-2020 (untreated, sham- and angiotensin II (AngII)-treated mice) [14], and Zebrafish-Ma-2021 (uninjured (control) vs. injured zebrafish hearts) [21]. ExpressHeart consists of eight main panels (Figure 1B). The “Home” panel introduces the web portal and the datasets included. Panels 2–6 correspond to pre-conducted analysis results of the five datasets. In each panel, corresponding to each dataset, ExpressHeart shows uniform manifold approximation and projection (UMAP) [22] and t-distributed stochastic neighbor embedding (t-SNE) [23] visualizations for cells of all cell types, and displays the expression levels of the user’s genes of interest by violin plot, feature plot, and heatmap. Differentially expressed genes (DEGs) for major cell types/subtypes are listed in tables. In the “Cross-comparison’’ panel, we highlight the DEG query across datasets, provide high confidence DEGs, and conduct cross-species comparisons among humans, mice, and zebrafish under normal conditions. In the “Download” panel, users can easily download all DEGs tables generated in the three analyses panels. Finally, the “Help” panel provides basic instructions to use this web portal.

### 2.2. Datasets in ExpressHeart

ExpressHeart currently consists of five scRNA-seq datasets of nonCMs from three species, namely humans, mice, and zebrafish (Figure 1A; Table 1). Specifically, we have one human dataset, two mouse datasets, and one zebrafish dataset. Note, the Human-Hocker-2021 are generated using snRNA-seq, whereas the rest are scRNA-seq. All five datasets were produced by the 10x Genomics Chromium platform. More details on the numbers of cells of each cell type and subtypes for each dataset can be found in Supplementary Tables S1 and S2.

1. The Human-Hocker-2021 dataset comprises 8993 nonCM nuclei from the hearts of two healthy adult human donors. Nine cell types were identified, including fibroblasts, myofibroblasts, endothelial cells, pericytes, adipocytes, smooth muscle cells, nerve cells, and two groups of immune cells (macrophages and lymphocytes).
2. The Mouse-Wang-2021 dataset consists of 12,779 cells from two adult mice, encompassing six major nonCMs cell types, namely fibroblasts, endothelial cells, pericytes, and three types of immune cells (macrophages, granulocytes, and lymphocytes). Of them, the three major cell types (fibroblasts, endothelial cells, and macrophages) are further clustered into three to five subtypes (Supplementary Table S2), with each subtype representing a distinct functional state.
3. The Mouse-Farhebi-2019 dataset comprises of 12,991 nonCM cells from control and injured mouse hearts (3 and 7 days post-MI surgery), where 5658, 3825, and 3508 cells are from healthy, 3 days post-MI and 7 days post-MI hearts, respectively. Unbiased clustering identified 24 cell populations, including the major cell types, fibroblasts, endothelial cells, mural cells, and immune cells (macrophages, monocytes, dendritic cells, B cells, T cells, and natural killer (NK) cells). Similarly, there are multiple subtypes identified for the major cell types. For example, four subtypes are identified in fibroblasts, three in endothelial cells, and eight in macrophages/monocytes (Supplementary Table S2).
4. The Mouse-McLellan-2020 dataset comprises of 13,176 nonCM cells from four untreated, four sham- and eight AngII-treated adult mice, where AngII-treatment could stimulate pathological remodeling of the heart. A total of 14 cell types were identified, including fibroblasts, epicardial cells, endothelial cells, lymphatic endothelial cells, endocardial cells, smooth muscle cells, pericytes, Schwann cells, proliferating mesenchymal cells, macrophages, dendritic-like cells, granulocytes, B cells, and T/NK cells. In addition, nine, three, four, and two subtypes were identified for cell types, fibroblasts, endothelial cells, macrophages, and smooth muscle cells, respectively (Supplementary Table S2).
5. The Zebrafish-Ma-2021 dataset consists of 25,972 cells from uninjured (control) and injured zebrafish hearts. The authors performed scRNA-seq on nonCMs isolated from the adult zebrafish hearts before and after injury to investigate cellular functions of nonCMs during heart regeneration. The study generated transcriptome profiles for 6550, 9373, 7018, and 3031 cells before the injury, and at 2, 7, and 14 days post-injury, respectively. Nine clusters were identified in the uninjured hearts, namely, fibroblasts, endothelial cells, thrombocytes, and four types of immune cells (macrophages, neutrophils, resident mesenchymal cells, and lymphocytes). Analysis was performed for each of the three major cell types, fibroblasts, endothelial cells, and macrophages, and further identified four, four, and five subtypes, respectively (Supplementary Table S2).

### 2.3. Visualization via UMAP and tSNE

**Figure 2:**
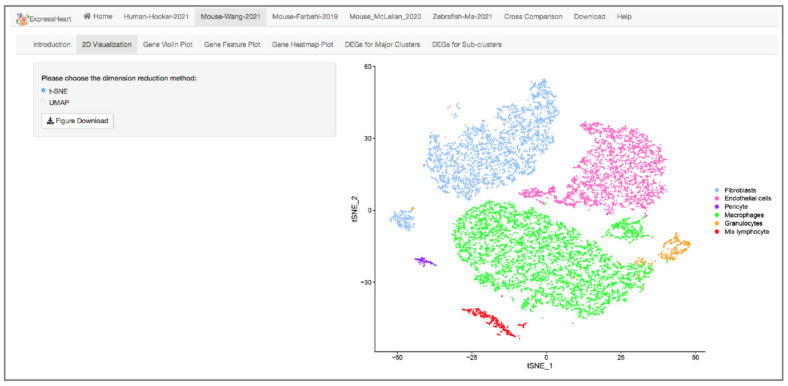
Interface of 2D visualization via UMAP/t-SNE for the Mouse-Wang-2021 dataset. On the left panel, users can choose different dimension reduction methods; the corresponding 2D UMAP/t-SNE plot is presented on the right. Each dot represents a cell/nucleus, and different colors correspond to different cell types.

ExpressHeart “2D Visualization” module provides visualizations for the cells from each dataset in a low dimensional space. Users can choose between UMAP and t-SNE, two widely used dimensional reduction and/or visualization methods, by selecting the corresponding checkbox at the left of the webpage (the left panel of Figure 2). In the plot, different cell types/clusters are shown in different colors (the right panel of Figure 2). Users can download the visualization figures simply by clicking the “Figure Download” button in the left panel (Figure 2).

### 2.4. Visualization of Gene Expression Levels via Violin Plot, Feature Plot, and Heatmap

**Figure 3:**
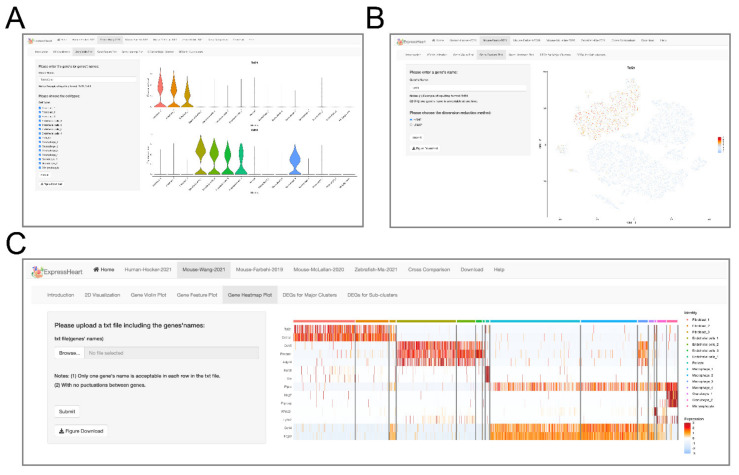
Interfaces of gene expression levels via (**A**) violin plot, (**B**) feature plot, and (**C**) heatmap. On the left panel of each interface, uses can specify a gene of interest for violin plot (**A**) or feature plot (**B**), or upload a text file containing a list of genes of interest for heatmap visualization (**C**). After clicking the “Submit” button, the corresponding violin/feature/heatmap plot for the target gene(s) will show on the right panel.

Our ExpressHeart allows users to check the expression levels for their genes of interest in three ways, providing important information from different perspectives to benefit investigators in the design and analysis of their studies, as well as in the interpretation of their results. First, ExpressHeart displays expression levels using violin plots, which allow users to easily compare the range and probability density of the expression of each gene across different cell types/subtypes. Users can type in the names of up to ten genes each time to generate the corresponding violin plot. We also allow users to select the cell types/subtypes of interest for the violin plot by simply checking the corresponding checkboxes. For example, in Figure 3A, the expression of gene *Tcf21* is shown by a violin plot across eight cell types/subtypes of the Mouse-Wang-2021 dataset. The generated plot can be downloaded by clicking the “Figure Download” button.

Second, ExpressHeart enables Feature Plot for visualizing the expression of a certain gene on the UMAP or t-SNE plot, which provides a more comprehensive view of the distribution of gene expression across cell types. For the plot, the gene expression level is illustrated using a gradient of colors from blue to red, whereby blue corresponds to low expression value, and red denotes high expression value. As shown in Figure 3B, gene *Tcf21* is shown to be exclusively highly expressed only in the three subtypes of fibroblast in the adult mouse heart. For computational efficiency and speedy browsing experience, ExpressHeart only allows one gene each time for Feature Plot.

Finally, the heatmap is one of the most widely used visualization strategies to show the magnitude of gene expression across cells, cell types, or samples. ExpressHeart also allows users to visualize and compare the expression levels of genes across cells from multiple cell types/subtypes using heatmap. Users can upload a list of genes of interest, and select the cell types to show in the heatmap by checking the corresponding checkboxes. In the heatmap, each row represents a gene, and each column represents a cell. The order of rows in the output heatmaps is the same as the input gene list. Cells from the same cell type/subtype are grouped together. For more informative visualization, we add color bars at the very top to illustrate the cell type/subtype membership of each cell. We use the same coloring scheme as in UMAP/t-SNE visualization for consistency and easier comparison across various visualization results. Figure 3C shows a heatmap of 14 genes for cells from all the 15 subtypes in the Mouse-Wang-2021 dataset, with the cells ordered by cell type/subtype, from the leftmost 2109 cells of fibroblast subtype 1 (Fibroblast_1; marked as red at the top bar) to the rightmost 360 cells of lymphocytes (marked as purple at the top bar). We observe clear block structures in the heatmap, allowing us to easily identify genes differentially expressed across cell types.

### 2.5. Browsing and Exporting DEGs

**Figure 4:**
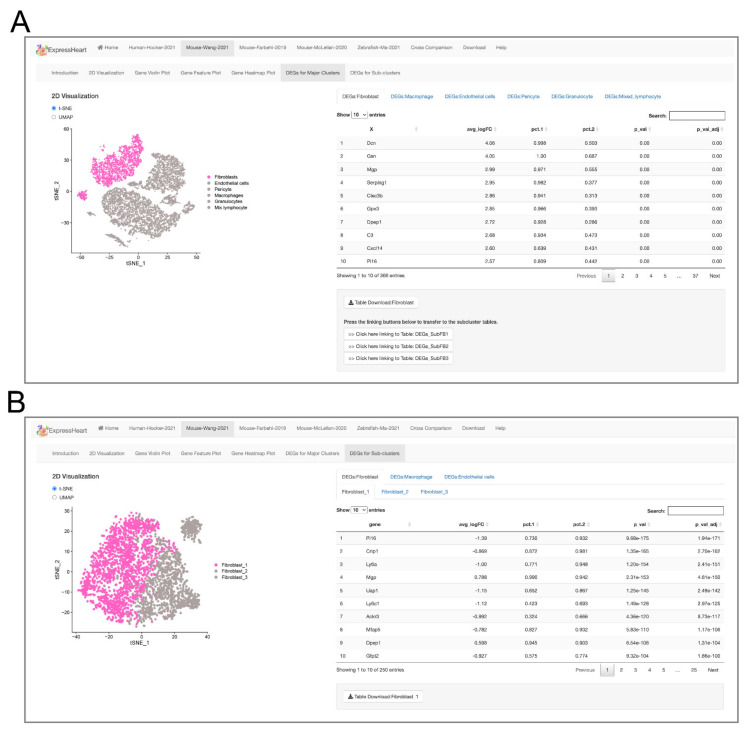
Interfaces of DEG lists for (**A**) each major cell type versus the other cell types, and (**B**) each subtype versus the other subtypes the same major cell type. On the left panel of each interface, the UMAP/t-SNE plot highlighting the target major cell type (**A**) or subtype (**B**) is presented, and the corresponding DEGs are listed in a table on the right.

For each dataset, ExpressHeart provides differential expression analysis among different cell types, as well as among different subtypes within each of the three major cell types, namely, fibroblasts, endothelial cells, and macrophages. DEGs are listed in tables (shown in Figure 4). Specifically, as shown in Figure 4A, we present DEGs in each cell type (versus the other cell types) in the right panel, and display the UMAP/t-SNE plot highlighting the target cell type in color at the left (the other cell types are in grey). Furthermore, we also present the DEGs for each subtype (versus other subtypes of the same major cell type) in a similar manner, where, in the UMAP/t-SNE plot of a major cell type, for example, fibroblast, the subtype of interest is highlighted in color, and other subtypes are in grey (Figure 4B). Note that all the tables of DEGs from ExpressHeart are available at the “Download” module.

### 2.6. Applications of ExpressHeart-Cross-Dataset Comparison

ExpressHeart incorporates multiple datasets from three species, thus allowing users to perform the analyses of nonCMs features between different datasets from the same species, as well as across different species. These analyses can provide a comprehensive view of the similarities and differences in the transcriptomic dynamics among different conditions or different species. Here we present four application examples of ExpressHeart.

#### 2.6.1. DEG Query

In ExpressHeart, we provide a panel “DEG Query”, allowing users to check whether a gene of interest is differentially expressed in any of the five datasets. For a given gene, ExpressHeart would search it against the DEG list of each cell type from each dataset, and list all the relevant information, including differentially expressing in which cell type and dataset, log-scaled fold change, the proportions of cells where the gene was detected in the target and background cell groups, and the adjusted *p*-value. The information is identical to the DEG information under each dataset, except that here homologs from other species will also be shown when searching a gene of interest. This function would allow users to rapidly obtain a comprehensive summary of DEGs in the existing datasets of the three species.

#### 2.6.2. High Confidence DEGs

**Figure 5:**
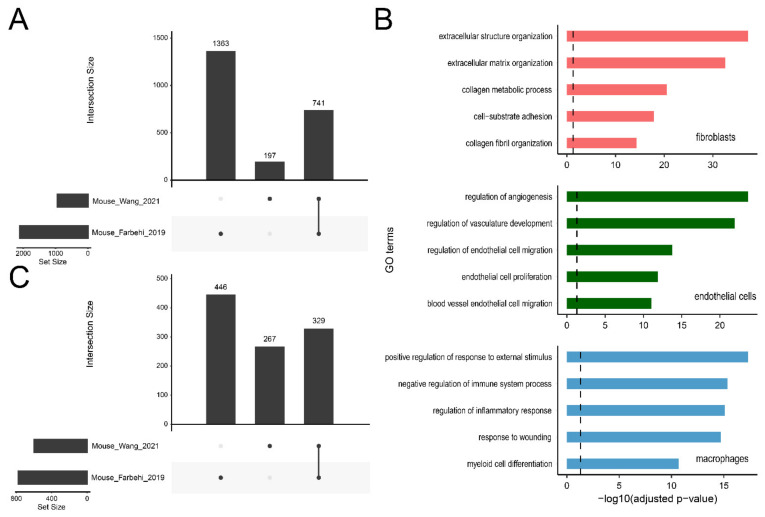
Upset plots of DEGs for three cell types combined (**A**) and fibroblast (**C**) between Mouse-Wang-2021 and Mouse-Farbehi-2019 datasets. Here, a total of 741 high confidence DEGs are detected for three cell types (fibroblasts, endothelial cells, and macrophages), including 329 DEGs for fibroblast. (**B**) Feature GO terms enriched for the high confidence DEGs of fibroblasts (upper panel), endothelial cells (middle panel), and macrophages (bottom panel). The dashed line represents the significance threshold of −log10(0.05).

For scRNA-seq data, cluster annotation largely depends on prior knowledge of the expression profiles of cell type-specific features. A comprehensive list of genes differentially expressed among cell types can improve the accuracy of cell type discovery. However, although there are a large amount of scRNA-seq data available, different datasets are generally generated from different laboratories, using different techniques, and/or under different conditions, and the DEGs identified in different datasets are usually different. With the help of ExpressHeart, we can obtain a confident list of DEGs for a certain cell type across multiple datasets. Between two mice datasets (Mouse-Wang-2021 and Mouse-Farbehi-2019), 741 shared DEGs are identified for three major cell types, fibroblasts, endothelial cells, and macrophages (Figure 5A). Gene ontology (GO) enrichment results show that these shared DEGs are highly relevant to the biological functions of the corresponding cell type (Figure 5B), where those identified in fibroblasts are enriched for extracellular matrix and collagen metabolism, and those in endothelial cells and macrophages are overrepresented in angiogenesis regulation and immune response, respectively. Moreover, we also identified 329 DEGs for fibroblasts subtypes shared between two mouse datasets (Figure 5C). These DEGs of high confidence can help to improve the accuracy of cell type annotation and discovery in scRNA-seq data.

#### 2.6.3. Matching Cell Subtypes across Datasets

**Figure 6:**
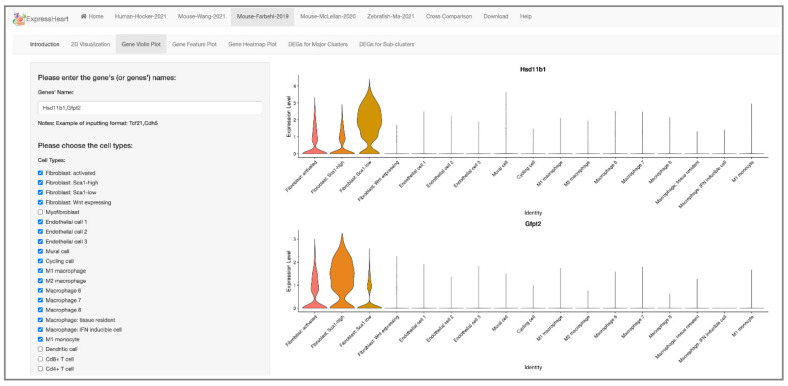
Comparison between two mice datasets. The two violin plots show the expression profiles of the feature genes of two fibroblast subtypes (*Hsd11b1* and *Gfpt2*, respectively) in the Mouse-Wang-2021 dataset in the major subtypes identified in the Mouse-Farbehi-2019 dataset. Users can type in the name(s) of target gene(s) and choose the cell type(s) of interest by checking the corresponding box(es) on the left panel, and the violin plot(s) showing the gene expression level will be presented on the right.

In general, different studies might identify subtypes for the major cell types with customized labels. Matching subtypes across datasets would facilitate the investigation of their biological functions. By comparing similarities in the expression profiles of subtype-specific features, ExpressHeart allows users to match subtypes across different datasets. Wang et al. identified three fibroblast subtypes with different functional states [18]. The two major subtypes, namely, FB1 and FB2, participate in cellular response and cytoskeleton organization.FB1 and FB2 are distinguished by several specific markers, for example, *Hsd11b1*, *Inmt* and *Cxcl14* for FB1, and *Gfpt2*, *Pi16*, and *Uap1* for FB2. Here, using ExpressHeart, we investigate the expression profiles of the six feature markers of the two fibroblast subtypes in the Mouse-Farhebi-2019 dataset to assess the potential biological roles of FB1 and FB2 subtypes during the MI process. As shown in Figure 6 and Supplementary Figure S1, the three FB1-feature markers *Hsd11b1*, *Inmt*, and *Cxcl14* are highly expressed in the FB1 subtype with low expression of *Sca1* (Fibroblast: Sca1-low in Figure 6), while the FB2-feature markers *Gfpt2*, *Pi16*, and *Uap1* are highly expressed in the FB2 subtype with high *Sca1* expression (Fibroblast: Sca1-high). These suggest that FB1 identified by the Mouse-Wang-2021 dataset corresponds to *Sca1*-low-expressing fibroblasts of the Mouse-Farhebi-2019 dataset, and FB2 corresponds to *Sca1*-high-expressing fibroblasts. Fibroblast: *Sca1*-low-expressing fibroblasts were found to transit into *Wnt*-expressing fibroblasts, promoting the differentiation of monocytes during heart repair in response to MI [11]. It is consistent with the proposed function of FB1 in cellular response [18].

#### 2.6.4. Cross-Species Distribution Comparison

Although the fundamental physiological processes in hearts are conservative across species, there are still some genes playing different roles in different species. For example, both under injury conditions, there are 391 homologs identified to be differentially expressed in the three major cell types of both mice and zebrafish, while 414 and 1363 homologs are exclusively differentially expressed in mice and zebrafish, respectively (Supplementary Figure S2). Therefore, characterizing and comparing in transcriptional dynamics of homolog genes across different species can decode their similar or different functional roles.

**Figure 7:**
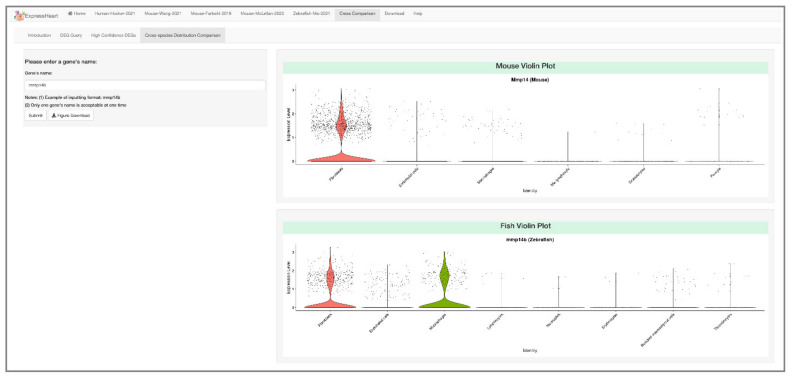
Cross-species comparison between mouse and zebrafish datasets. Users can input the homolog gene name on the left panel, and the corresponding violin plots for the homolog genes (*mmp14b* in zebrafish, and *Mmp14* in mouse) are shown on the right. Here, *mmp14b* is expressed in 28.0% and 27.8% of fibroblasts and macrophages of zebrafish, while its homolog gene *Mmp14* is expressed in a similar proportion of fibroblasts (28.5%), but in a much lower proportion of macrophages (0.9%) of mouse.

Furthermore, ExpressHeart can also visualize the expression profiles of a given gene across the three species under the normal/healthy condition. In Figure 7, we present an example: *mmp14b* is expressed in zebrafish in both fibroblasts and macrophages with proportions 28.0% and 27.8%, respectively. In contrast, *Mmp14* in mice is expressed in a similar proportion of fibroblasts (28.5%), but in a much lower proportion of macrophages (0.9%). Thus, with ExpressHeart, users can get a clear and more comprehensive view of both the similarities and the differences in gene expression profiles across species.

## 3. Discussion

An increasing amount of scRNA-seq data has been generated for nonCMs; however, there is currently no method or platform allowing researchers to easily visualize or analyze these datasets. To address this issue, we developed ExpressHeart, which is a web server for visualizing and analyzing scRNA-seq data from public nonCMs datasets under various conditions and from two different species. Specifically, ExpressHeart incorporates three scRNA-seq data from (1) healthy hearts from adult human donors, (2) healthy/normal hearts from adult mice, (3) control and MI injured hearts from mice, and (4) control/uninjured and injured hearts of 2, 7 and 14 days post-injury from zebrafish (Figure 1A). ExpressHeart provides UMAP/t-SNE visualization, shows expression profiles across cell types/subtypes for genes of interest via feature plot, violin plot, and heatmap plot, tabulates DEGs in a certain cell type/subtype, and provides cross-comparisons between different datasets, as well as different species (Figure 2, Figure 3, Figure 4, Figure 5, Figure 6 and Figure 7). By providing the conveniently displayed results, ExpressHeart not only aids investigators to gain a more comprehensive understanding of the functions of nonCMs in different physiological conditions (in healthy or MI conditions, or heart regeneration processes), but also provides important information that can guide the experimental design and analysis of future studies.

ExpressHeart provides an interface to directly compare transcriptional profiles across different datasets. On the one hand, for the same species, ExpressHeart provides users lists of high confidence cell-type-specific DEGs by taking the intersection to DEGs obtained from multiple datasets. These high confidence DEGs could unify cell type identification, and thus, increase the reproducibility of biological findings. Furthermore, we could also use the high confidence DEGs to match the corresponding cell subtypes across studies, which can help us distinguish different biological functions for distinct subtypes and potentially lead to the identification of new subtypes.

On the other hand, ExpressHeart allows cross-species distribution comparison of nonCMs features among humans, mice, and zebrafish, which can provide a comprehensive view of the similarities and differences in the transcriptomic profiles of homolog genes across those three species. One example is shown in Figure 7. In mice, *Mmp14* encodes the endopeptidase involved in extracellular matrix component degeneration, and is expressed only in a high proportion of fibroblasts (28.5%), while in zebrafish, its homolog *mmp14b* is found to be expressed in both fibroblasts (28.0%) and macrophages (27.8%), indicating it may also play a role in the immune process. This example suggests that, although certain regulatory pathways are shared across species, there are differences between mice and zebrafish. A better understanding of these differences could provide new insights into the differentiation in heart growth and injury response processes across different species.

In the current version, ExpressHeart mainly focuses on visualizing and characterizing the molecular features of nonCMs profiled by scRNA-seq. With the rapid development of omics technologies, many other single-cell assays have become available to measure other aspects of the cell features beyond gene expression signatures, such as DNA methylation [24], open chromatin status [25,26], and chromatin interactions [27]. Several studies have profiled the specific open chromatin landscapes controlling the cellular identity of nonCMs [17,18]. For example, using a single-nucleus assay for transposase-accessible chromatin using sequencing (snATAC-seq), Hocker et al. identified more than 280,000 cis-regulatory elements (CREs) in the hearts of healthy adult humans, and found that cell-type-specific CREs and their trans-acting factors are associated with cardiac structures, functions and disease pathogenesis [17]. In addition, the different activities of distinct CREs and trans-acting factors are also found to contribute to the differentiation among subtypes [18]. Therefore, further work would involve the incorporation of multi-omics single-cell assays to obtain a comprehensive view of the molecular features of nonCMs beyond the transcriptomic profile.

## 4. Materials and Methods

### 4.1. Processing of scRNA Sequencing Datasets

The five datasets presented in ExpressHeart were pre-processed and analyzed following the pipeline described in the corresponding publications.

#### 4.1.1. Human-Hocker-2021

For the Human-Hocker-2021 dataset, the expression matrix of 24,535 high-quality cells was downloaded from Gene Expression Omnibus (GEO) with accession number GSE165838. These cell nuclei were isolated from four cardiac chambers (right and left atrium, and right and left ventricles) of four healthy donors. Following the analytic pipeline described in Hocker et al. [17], the expression counts of each cell were scaled by the total counts and a scaling factor of 10,000 and natural-log normalized using the *NormalizeData* function of Seurat v3 [28]. The top 3000 highly variable genes (HVGs) were identified by the *FindVariableFeatures* function, and then all the genes were scaled and centered by the *ScaleData* function. Principal components analysis (PCA) was performed using the *RunPCA* function with the 3000 HVGs as input. To remove the batch effect between different donors, the *RunHarmony* function from the Harmony package [29] was employed with the first 30 principal components (PCs) to merge cells from the two donors. We computed t-SNE and UMAP coordinates using the first 14 Harmony components by the *RunTSNE* and *RunUMAP* functions of Seurat v3 [28], respectively. Cell identities were assigned according to the annotations from Hocker et al. [17]. Only major cell types were included in the downstream analysis. To speed up for ExpressHeart, we downsampled each of the four large cell types, fibroblasts, endothelial cells, pericytes, and macrophages, to 1500 cells, and kept all 2993 cells from five rarer cell types (including smooth muscle cells, myofibroblasts, adipocytes, nerve cells, and lymphocytes), leading to a total of 8993 remaining cells (Supplementary Table S1).

#### 4.1.2. Mouse-Wang-2021

For the Mouse-Wang-2021 dataset, single live nonCM cells were isolated from the left and right ventricles of adult mice and transcriptionally profiled using the 10× Genomics Chromium platform (10× Genomics, Inc., Pleasanton, CA, USA). Low-quality cells expressing <200 genes and genes expressed in less than three cells were first filtered out from each of the two biological replicates. For each sample, the top 2000 HVGs were identified. Then, the two biological replicates were merged and corrected for batch effect using Seurat v3 [28]. Data dimensions were reduced by PCA, and unsupervised clustering was performed to identify cell clusters using the *FindClusters* function, with the first 20 PCs and the resolution parameters set to 0.5. The obtained clusters were defined according to the expression levels of known cell-type-specific markers. Since we only focused on nonCMs, cluster(s) highly expressing canonical markers of cardiomyocytes were removed from downstream analysis.

#### 4.1.3. Mouse-Farbehi-2019

For the Mouse-Farbehi-2019 dataset, the expression matrix of 15,073 cells, which were isolated from ventricles and interventricular septum, was downloaded from EMBL-EBI with accession number E-MTAB-7376. Data analysis was carried out in Seurat v2 [30]. Specifically, cells with <200 genes expressed, or <500 unique molecular indices (UMIs), or >5% RNA mapped to mitochondrial genes were first removed. Genes expressed in less than ten cells were also filtered out. Furthermore, cells with >4000 expressed genes or 20,000 UMIs were considered potential doublets and excluded from further analysis. Variations, due to the total number of UMIs, were regressed out during the data scaling process, using the *ScaleData* function. PCA was performed, and the first 54 PCs were selected for unsupervised clustering with a resolution of 1.2.

#### 4.1.4. Mouse-McLellan-2020

For the Mouse-McLellan-2020 dataset, the expression matrix of 29,615 cells, isolated from cardiac ventricles of untreated, sham- and Ang II-treated mice, was downloaded from EMBL-EBI with accession number E-MTAB-8810. Cells with <100 or >15,000 genes expressed, or >50,000 UMIs or >30% RNA mapped to mitochondrial genes were filtered out. Genes expressed in <10 cells were also excluded from the dataset. Analysis was performed in Seurat v3 [28]. Top 2000 HVGs were detected, and PCA was performed. The first 30 PCs were selected for unsupervised clustering with a resolution of 1.2. Cluster(s) highly expressing cardiomyocyte markers were removed from downstream analysis. To speed up ExpressHeart, we downsampled each of the 13 large cell clusters to 700 cells, while kept all cells from 15 rarer cell clusters, leading to a total of 13,176 remaining cells (Supplementary Table S1).

#### 4.1.5. Zebrafish-Ma-2021

For the Zebrafish-Ma-2021, low-quality cells, i.e., those expressing <200 genes, were first removed. Cells from each sample were clustered using Seurat v2 [30]. Clusters highly expressing cardiomyocyte markers, including *tnnt2*, *ckma*, and *nppa*, were filtered out. In addition, clusters that express a high level of typical markers of any two cell types and are of observed frequencies close to the expected values were considered doublets and excluded from downstream analysis. All the cells retained from all four-time points were merged using LIGER v0.3.1 [31]. Briefly, HVGs with variance > 0.1 were selected for each sample, and the union was taken for the subsequent analysis. Factorization was performed on the scaled data using the *optimizeALS* function, with the number of factors (*k*) set to 45. All the samples were merged using the *quantileAlignSNF* function with the resolution parameter set to 2. After alignment, nonspecific factors (technical factors), consisting of mitochondrial, ribosomal, or cell cycle genes, were excluded from downstream analysis. In the merged data, potential doublets were also filtered out.

### 4.2. Data Visualization

For each of the five datasets, the dimensionality of the scRNA-seq data was reduced by two methods, UMAP, and t-SNE, for visualization using the *Dimplot* function of the Seurat package. For gene(s) provided by users, feature plot, violin plot, and heatmap are produced using the *FeaturePlot*, *VlnPlot*, and *DoHeatmap* functions of Seurat v3 [28].

### 4.3. Analysis of DEGs

Differential expression analysis was performed among different nonCM cell types, in each of the five datasets presented in ExpressHeart. In addition, DEGs among the subtypes of the three major cell types (fibroblasts, endothelial cells, and macrophages) in each of the two mouse datasets and the zebrafish dataset were also detected and presented in ExpressHeart. Specifically, DEGs in a certain cell type/subtype were identified by comparing them to cells of the other cell types/subtypes using the Wilcoxon Rank Sum test by the *FindMakers* function of Seurat v3 [28]. Genes (1) expressed in a minimal 25% of cells in the target cell types/subtypes, (2) 1.28-fold higher (upregulated) or lower (downregulated) than the rest of cells, and (3) with adjusted *p*-values < 0.05 are considered as significantly DEGs in that cell types/subtypes.

### 4.4. High Confidence DEGs

We defined high confidence DEGs as those shared across multiple (≥2) datasets in the same species. We first compared DEGs for three major cell types (namely fibroblasts, endothelial cells, and macrophages) across two mouse datasets. We generated upset plots to show the numbers of high confidence DEGs and the number of dataset-specific DEGs. To assess the functional relevance of the DEGs, we performed GO enrichment analysis for DEGs of the three major cell types that are identified in both Mouse-Wang-2021 and Mouse-Farbehi-2021 datasets, using ClusterProfiler [32]. GO terms with adjusted *p*-value < 0.05 were considered as significantly overrepresented.

### 4.5. Cross-Species Distribution Comparison

For the cross-species distribution comparison, we generated gene violin plots to compare the expression distribution in each cell type for each species. For mouse and zebrafish, the uninjured zebrafish cells from the Zebrafish-Ma-2021 dataset were first extracted, and then compared with the wildtype mouse cells from the Mouse-Wang-2021 dataset. The lists of homolog genes among any two of the three species—humans, mice, and zebrafish, was downloaded from ensembl BioMart. Only genes having homologs across those three species (or between any of two) are visualized in ExpressHeart.

### 4.6. Web Server Implementation

ExpressHeart consists of a web frontend application and a backend database. The frontend web, displaying visualization and results, is constructed in R Shiny [33]. Our R Shiny application mainly contains two parts: User interaction (UI) and server backend. Seven functional panels are created in the web frontend, including two descriptive panels: “Home” and “Help”; five data analysis panels corresponding to five datasets from three species: “Human-Hocker-2021, “Mouse-Wang-2021”, “Mouse-Farbehi-2019” and “Zebrafish-Ma-2021”; one cross-dataset-comparison panel: “Cross-comparison”; and one “Download” panel to download DEGs. When the server backend receives requests/input from the web user via the web frontend, it loads the database required for the analysis, generates corresponding figures, and tables, and transmits the results to the web frontend. Notably, for efficiency, some analysis results (figures and tables produced without the requirement to user’s input) are kept static in that they have been already deposited at the backend and can be directly transmitted to the web frontend.

## 5. Conclusions

In summary, we present ExpressHeart, a comprehensive visualization and analysis web portal for nonCM cells in mice and zebrafish. ExpressHeart is a valuable resource for investigators studying molecular mechanisms of cardiac homeostasis and repair. ExpressHeart is freely available at: http://shiny.bios.unc.edu/expressheart/.

## Figures and Tables

**Table 1:** Datasets incorporated in ExpressHeart. All three datasets are generated from 10× Genomics Chromium.

Dataset	Species	Technologies	Condition	Cells/Nuclei	Genes	Ref
Human-Hocker-2021	Human	snRNA-seq	Healthy	8993	27,109	[17]
Mouse-Wang-2021	Mouse	scRNA-seq	Wildtype	12,779	27,998	[18]
Mouse-Farbehi-2019	Mouse	scRNA-seq	Control vs. MI	12,991	15,674	[11]
Mouse-McLellan-2020	Mouse	scRNA-seq	Untreated, sham- and Ang II-treated	13,176	17,170	[14]
Zebrafish-Ma-2021	Zebrafish	scRNA-seq	Control vs. Injury	25,972	35,117	[21]

## Data Availability

ExpressHeart is freely available at: http://shiny.bios.unc.edu/expressheart/. In ExpressHeart, we adopted five datasets: (1) scRNA-seq dataset of nonCMs from hearts of two healthy Adult human donors from Hocker et al. [17] (GEO accession number GSE165838), (2) scRNA-seq dataset of nonCMs from normal adult mouse hearts from Wang et al. [18] (GEO accession number GSE157444), (3) snRNA-seq dataset of nonCM cells from control and injured mouse hearts (3 and 7 days post-MI surgery) from Farbehi et al. [11] (EMBL-EBI accession number E-MTAB-7376), (4) scRNA-seq dataset from the hearts of untreated, sham- and AngII-treated mice from McLellan et al. [14] (EMBL-EBI accession number E-MTAB-8810), and (5) scRNA-seq dataset of nonCMs isolated at multiple time points during zebrafish heart regeneration from Ma et al. [21] (GEO accession number GSE145980).

## Supplementary Materials

The following are available online at https://www.mdpi.com/article/10.3390/ijms22168943/s1. Supplementary Table S1: Cell number and proportion for each major cell type in the five datasets presented in ExpressHeart, Supplementary Table S2: Cell number and proportion for the subtypes of the major cell types in three datasets presented in ExpressHeart, Supplementary Figure S1: Violin plots showing the expression profiles of the feature genes of fibroblast subtypes, Supplementary Figure S2: Upset plots of homolog DEGs for three major cell types combined between Zebrafish-Ma-2021 and Mouse-Farbehi-2019 datasets.
