# Supplementary material for: ExpressHeart: Web Portal to Visualize Transcriptome Profiles of Non-Cardiomyocyte Cells
Source: Int J Mol Sci. 2021 Aug 19;22(16):8943. doi: 10.3390/ijms22168943 (PMC8396223; doi:10.3390/ijms22168943)
Supplement: Supplementary file 1 [file ijms-22-08943-s001.zip › ijms-1320236-supplementary.pdf]

Supplementary Table S1. Cell (nuclei) number and proportion for each major cell type in the four datasets presented in ExpressHeart.

a. Nuclei number and proportion for each cell type in the Human-Hocker-2021 dataset

	Number of nuclei before downsampling	Number of nuclei	Proportion (%)
Fibroblasts	10,577	1500	16.68
Endothelial cells	6250	1500	16.68
Pericytes	2962	1500	16.68
Macrophages	1753	1500	16.68
Myofibroblasts	1012	1012	11.25
Smooth muscle cells	669	669	7.44
Adipocytes	525	525	5.84
Nerve cells	465	465	5.17
Lymphocytes	322	322	3.58

b. Cell number and proportion for each cell type in the Mouse-Wang-2021 dataset

	Number of cells	Proportion (%)
Fibroblasts	3459	27.07
Endothelial cells	2939	23.00
Pericyte	125	0.98
Macrophages	5532	43.29
Granulocytes	364	2.85
Lymphocytes	360	2.82

c. Cell number and proportion for each cell type in the Mouse-Farbehi-2019 dataset

	Number of cells	Proportion (%)
Fibroblasts	3643	28.04
Myofibroblasts	355	2.73
Endothelial cells	2648	20.38
Mural cells	227	1.75
Cycling cells	182	1.40
M1 monocytes	606	4.66
Macrophages	3538	27.23
Dendritic cells	384	2.95
T cells	562	4.33
B cells	748	5.76
NK cells	74	0.57
Glial cells	24	0.18

d. Cell number and proportion for each cell type in the Zebrafish-Ma-2021 dataset

	Number of cells	Proportion (%)
Fibroblasts	6770	26.07
Endothelial cells	8554	32.94

Macrophages	6808	26.21
Lymphocytes	768	2.96
Erythrocytes	1032	3.97
Resident mesenchymal cells	1647	6.34
Neutrophils	187	0.72
Thrombocytes	206	0.79

e. Cell number and proportion for each cell type in the Mouse-McLellan-2020 dataset

	Number of cells before downsampling	Number of cells	Proportion (%)
Fibroblasts	15,753	5526	41.94
Macrophages	5002	2322	17.62
Endothelial cells	3762	1705	12.94
Smooth muscle cells	1043	796	6.04
Endocardial cells	1295	700	5.31
Pericytes	411	411	3.12
B cells	325	325	2.47
T/NK cells	279	279	2.12
Granulocytes	265	265	2.01
Schwann cells	261	261	1.98
Lymphatic endothelial cells	253	253	1.92
Epicardial cells	127	127	0.96
Dendritic-like cells	119	119	0.90
Proliferating mesenchymal cells	87	87	0.66

Supplementary Table S2. Cell number and proportion for the subtypes of the major cell types in three datasets (Mouse-Wang-2021, Mouse-Farbehi-2019 and Zebrafish-Ma-2021) presented in ExpressHeart.

a. Cell number and proportion for the subtypes of the major cell types in the Mouse-Wang-2021 dataset

	Number of cells	Proportion (%)
**Fibroblasts**		
Fibroblast_1	2109	60.97
Fibroblast_2	1134	32.78
Fibroblast_3	216	6.24
**Endothelial cells**		
Endothelial cells_1	2040	69.41
Endothelial cells_2	626	21.30
Endothelial cells_3	205	6.98
Endothelial cells_4	68	2.31
**Macrophages**		
Macrophage_1	3089	55.84
Macrophage_2	1929	34.87
Macrophage_3	344	6.22
Macrophage_4	170	3.07
**Granulocytes**		
Granulocyte_1	54	14.84
Granulocyte_2	310	85.16

b. Cell number and proportion for the subtypes of the major cell types in the Mouse-Farbehi-2019 dataset

	Number of cells	Proportion (%)
**Fibroblasts**		
Fibroblast: activated	1049	28.79
Fibroblast: Sca1-high	826	22.67
Fibroblast: Sca1-low	1650	45.29
Fibroblast: Wnt expressing	118	3.24
**Endothelial cells**		
Endothelial cell 1	1874	70.77
Endothelial cell 2	372	14.05
Endothelial cell 3	402	15.18
**Macrophages**		
M1 macrophage	2078	58.73
M2 macrophage	624	17.64
Macrophage: tissue resident	236	6.67
Macrophage: IFN inducible cell	155	4.38
Macrophage 6	169	4.78
Macrophage 7	141	3.99
Macrophage 8	135	3.82
**T cells**		
Cd8+ T cell	319	56.76

Cd4+ T cell	243	43.24

c. Cell number and proportion for the subtypes of the major cell types in the Zebrafish-Ma-2021 dataset

	Number of cells	Proportion (%)
**Fibroblasts**		
Fibroblasts_1	2546	37.61
Fibroblasts_2	2039	30.12
Fibroblasts_3	1739	25.69
Fibroblasts_4	446	6.59
**Endothelial cells**		
Endothelial cell_1	6877	80.40
Endothelial cell_2	1300	15.20
Endothelial cell_3	265	3.10
Endothelial cell_4	112	1.31
**Macrophages**		
Macrophage_1	2416	35.49
Macrophage_2	2027	29.77
Macrophage_3	1742	25.59
Macrophage_4	496	7.29
Macrophage_5	127	1.87

d. Cell number and proportion for the subtypes of the major cell types in the Mouse-McLellan-2020 dataset

	Number of cells before downsampling	Number of cells	Proportion (%)
**Fibroblasts**			
Fibroblasts: Thbs4	422	422	7.64
Fibroblasts 2	1813	700	12.67
Fibroblasts 3	1468	700	12.67
Fibroblasts 4	2133	700	12.67
Fibroblasts 5	3502	700	12.67
Fibroblasts 6	3316	700	12.67
Fibroblasts: Wif1	1436	700	12.67
Fibroblasts: Cilp	1332	700	12.67
Fibroblasts 9	204	204	3.69
**Endothelial cells**			
Endothelial cells 1	2583	700	41.06
Endothelial cells 2	305	305	17.88
Endothelial cells 3	874	700	41.06
**Macrophages**			
Macrophages 1	1120	700	30.15
Macrophages 2	1565	700	30.15
Macrophages 3	2095	700	30.15
Macrophages 4	222	222	9.55

**Smooth muscle cells**			
Smooth muscle cells 1	947	700	87.94
Smooth muscle cells 2	96	96	12.06

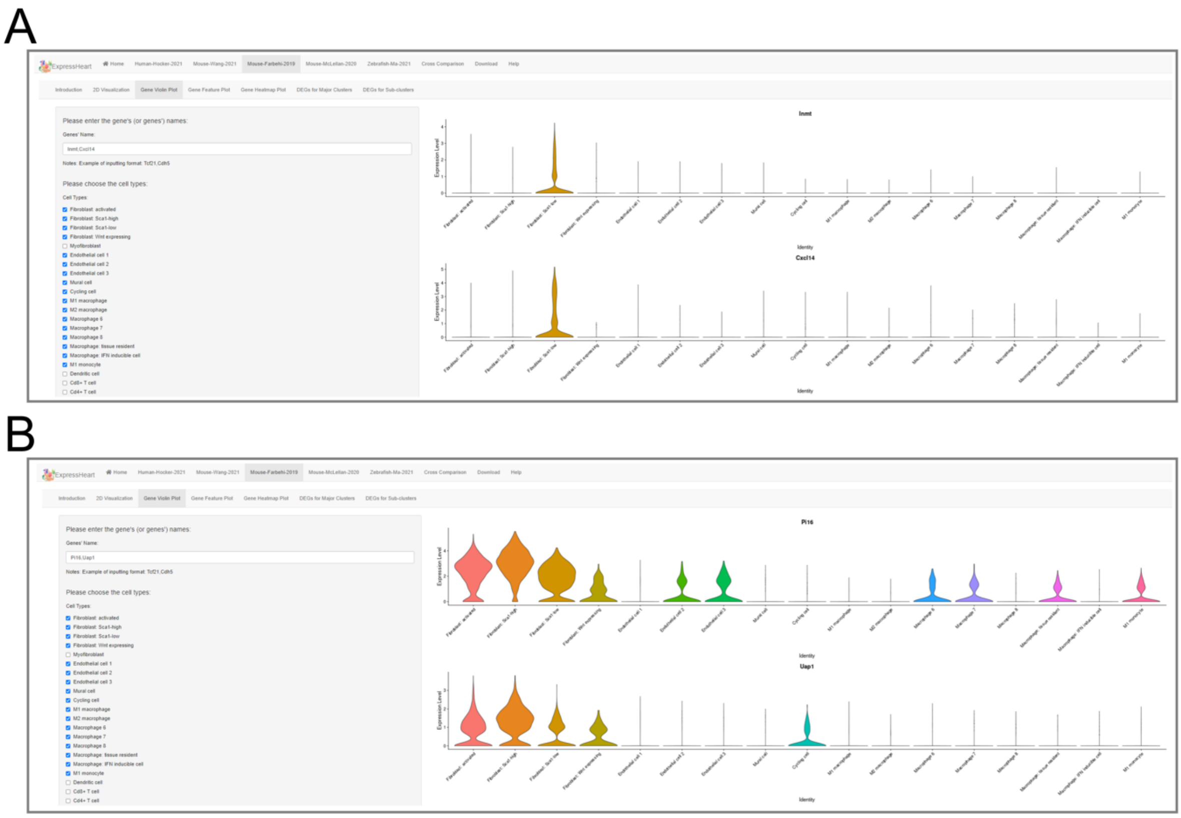

Supplementary Figure S1: Violin plots showing the expression profiles of the feature genes of fibroblast subtypes. Here we show the expression profiles of the feature genes of fibroblast subtype 1 (*Inmt* and *Cxcl14*) (**A**) and 2 (*Pi16* and *Uap1*) (**B**) in Mouse-Wang-2021 dataset in the major subtypes identified in Mouse-Farbehi-2019 dataset.

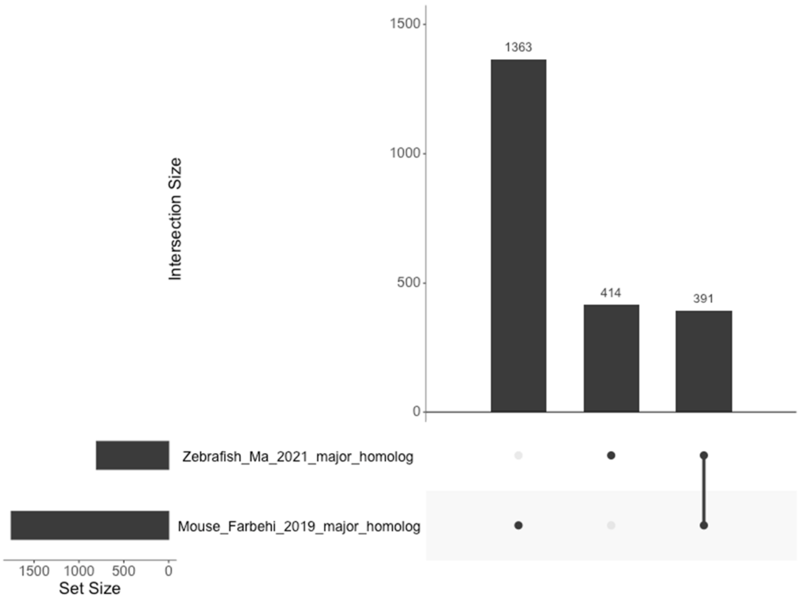

Supplementary Figure S2: Upset plots of homolog DEGs for three major cell types combined between Zebrafish-Ma-2021 and Mouse-Farbehi-2019 datasets.
